# Controlling the spectrum of photons generated on a silicon nanophotonic chip

**DOI:** 10.1038/ncomms6489

**Published:** 2014-11-20

**Authors:** Ranjeet Kumar, Jun Rong Ong, Marc Savanier, Shayan Mookherjea

**Affiliations:** 1 Department of Electrical and Computer Engineering, University of California, San Diego, La Jolla, California 92093, USA

## Abstract

Directly modulated semiconductor lasers are widely used, compact light sources in optical communications. Semiconductors can also be used to generate nonclassical light; in fact, CMOS-compatible silicon chips can be used to generate pairs of single photons at room temperature. Unlike the classical laser, the photon-pair source requires control over a two-dimensional joint spectral intensity (JSI) and it is not possible to process the photons separately, as this could destroy the entanglement. Here we design a photon-pair source, consisting of planar lightwave components fabricated using CMOS-compatible lithography in silicon, which has the capability to vary the JSI. By controlling either the optical pump wavelength, or the temperature of the chip, we demonstrate the ability to select different JSIs, with a large variation in the Schmidt number. Such control can benefit high-dimensional communications where detector-timing constraints can be relaxed by realizing a large Schmidt number in a small frequency range.

## Abstract (web summary)

The controlled creation of single and pair photon sources on a silicon chip is important for the realisation of quantum optical communications. Here, the authors control the spectrum of such photons generated on a silicon chip.

## Graphical abstract

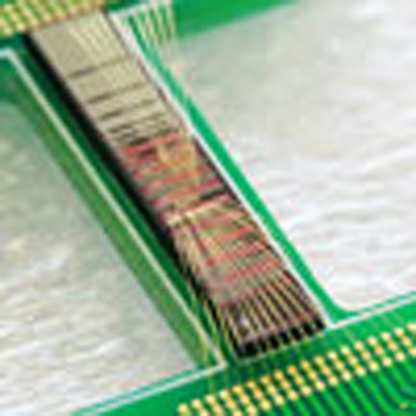

Modern communication systems seek to use more degrees of freedom to encode information than simple on–off keying^1^. When transferring information using individual or pairs of photons, it is especially important to maximize the number of bits per photon because of the ‘photon-starved’ nature of the communication system. Some of the conventional methods to encode information includes polarization-bin encoding^2^, time-bin encoding^3^ and frequency-bin encoding^4^. However, the quantum equivalent of manipulating the spectrum of the source has not been demonstrated. Although directly modulated semiconductor lasers are widely used and well understood, demonstrating a similar concept in quantum optics is not simple because a number of issues have to be simultaneously addressed. These include the following: showing a photon source using semiconductor technology that can be easily manufactured, designing internal degrees of freedom in the architecture of the source that can be externally manipulated by a user to generate different quantum spectra and developing a measurement procedure for the different photon spectra, rather than simply counting photons.

The photon-pair generation process used here was based on the spontaneous four-wave mixing (SFWM) nonlinear Kerr interaction, which generates a pair of photons (sometimes called the ‘signal’ and ‘idler’ photons by analogy with classical parametric mixing). Two photons from a single optical pump beam at frequency 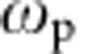 are absorbed by the semiconductor, and two photons are simultaneously generated at signal (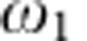) and idler (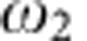) frequencies. Energy conservation requires that 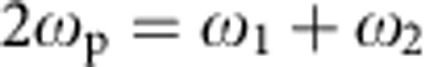, and momentum conservation (phase-matching) is also necessary for appreciable rates of pair production. The high SFWM nonlinearity in silicon photonic chips has been used in a number of demonstrations, including photon pairs of high quality, heralded single photons and spectral multiplexing^5^,^6^,^7^,^8^,^9^,^10^,^11^. Unlike quantum dots, these photon sources can operate at room temperature and can be coupled relatively easily to optical fibres. Using SFWM rather than spontaneous parametric down-conversion (SPDC) has the advantage that all the optical wavelengths are within a relatively narrow range near the telecommunications band centred at 1.55 μm, which simplifies the waveguiding and phase-matching requirements. In the reported experiments, the peak and average pump power required were only ~0.6 and 0.2 mW, respectively, considerably lower than used in bulk crystal-based SPDC sources and optical fibre-based SFWM sources of photon pairs. Lower average power can be achieved, for example, in SPDC using mode-locked lasers; however, the peak power requirements are significantly higher^12^. One of the potential problems of SFWM is broad-band Raman scattering in glass optical fibres, which is partially mitigated in crystalline silicon waveguides, in which spontaneous Raman scattering (SRS) is narrow band, and the SRS-shifted light may not coincide with one of the guided modes^13^.

Under the appropriate experimental conditions, the silicon device generates a two-photon state, with a small likelihood of undesirable states such as four-photon states, six-photon states and so on^7^,^13^. For the type of device studied here experimentally, which consists of coupled-microring resonators, we have previously identified the range of input powers over which pair generation with a high coincidence-to-accidental ratio can be achieved, and when one of the photons can be used as a herald, projecting the other into a single-photon state, with *g*^(2)^(0)≪0.2 (ref. 8). The bi-photon state |1›_1_|1›_2_ generated by SFWM is


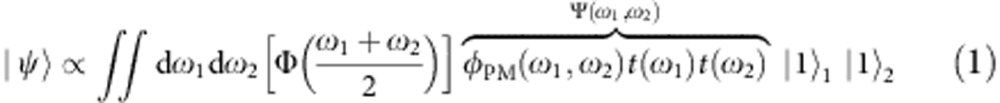


where *Φ* is the pump spectral envelope (typically taken as a Gaussian function^14^), φ_PM_ is the phase-matching function, and *t* is the transfer function (amplitude transmission) of the device. Conventionally (for example, in bulk crystals or fibres), *φ*_PM_=sinc(Δ*kL*) where *L* is the length of the waveguide and Δ*k* is the deviation from perfect phase-matching, Δ*k*=2*k*_*p*_−*k*_1_−*k*_2_, in terms of the wavenumbers at the pump (*k*_p_), signal (*k*_1_) and idler (*k*_2_) wavelengths. In the devices studied here (see Supplementary Note 1 and Supplementary Fig. 1), the expression for Δ*k* is more complicated, involving a discrete set of wavenumbers and terms that describe the reduction of group velocity (slow-light effects) at these wavelengths (see Supplementary Note 2).

The joint spectral intensity (JSI) is given by the magnitude-squared of the joint spectral amplitude, which is the product of *Φ* and Ψ defined in the integrand of equation (1), and can be measured experimentally. The shape of the JSI in the 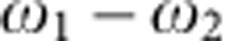 plane depends concurrently on the linewidth and wavelength of the pump, the phase-matching points and the transmission functions *T*_1_ and *T*_2_ in the passbands of the two generated photons. The JSI has been widely used in SPDC experiments for distinguishing between separable, correlated and anticorrelated two-photon states, and quantifying entanglement^12^,^15^,^16^,^17^,^18^,^19^,^20^,^21^,^22^,^23^,^24^,^25^,^26^. Manipulating the JSI for an SPDC source is a topic of active current research, mainly by manipulating the properties of the pump pulse^27^. Control over the JSI can be useful in creating a versatile SFWM pair source, whose emission properties can be adapted to the target application; for example, for a heralded single-photon source, uncorrelated photon pairs are needed 12,25,26,28, whereas for an entangled photon-pair source using the spectral degree of freedom, the photon pairs may need to be highly correlated^29^. Shaping the JSI of photon pairs after generation reduces their spectral brightness significantly, and it is beneficial instead to modify the optical density of states at the source^30^. Here we demonstrate a way to select the JSI of the generated photon pairs at the chip itself.

## Results

### Description of the JSI

**Figure 1:**
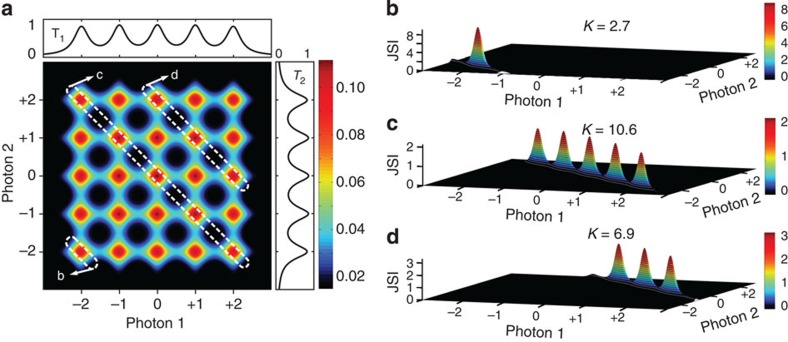
Controlling the JSI at the source. (**a**) In a five-resonator photon-pair source, this 2D plot obtained by numerical simulation shows the contours of the function Ψ defined in equation (1) as a function of the wavelengths of the two generated photons, relative to the band centre. Shown to the top and the right are the transmission amplitudes in the two bands. The number of transmission resonances in each band is equal to the number of resonators (five) and, together, they form 5 × 5=25 phase-matching points in the 2D plane at which photon-pair generation can be efficient. The wavelength of the pump determines which diagonally oriented slices of this phase-matching diagram comprise the JSI of the photon pair, with three particular possibilities for the JSI marked by regions ‘b’, ‘c’ and ‘d’. (**b**) The JSI of the two-photon state when the pump wavelength is positioned at the edge of its transmission band, showing a state with one major peak in the JSI. Such a state is suitable for heralding^17^,^28^. (**c**) The JSI when the pump wavelength is positioned in the middle of its transmission band, showing a state with five distinct peaks, which is suitable for an entangled pair source^17^,^29^. (**d**) The JSI when the pump is tuned to another resonance in its transmission band, with three peaks as an intermediate case. In each figure, the horizontal axes are in units of normalized wavelength (one unit equals the separation between adjacent peaks) measured relative to the band centre, and the vertical axes and colour scales are normalized so that the area under the JSI is unity. The *K* values represent the Schmidt numbers, that is, the dimensionality of the singular-value decomposition of the JSI.

Before discussing the experimental results, we first discuss theoretically how the JSI can be varied, and why the coupled-resonator structure is useful. In any pair source device, simultaneous energy-matching and phase-matching requirements determine the JSI of the two-photon state generated by a pump beam of a particular frequency. Our device consists of a periodic sequence of coupled microresonators, in which optical excitations propagate from input to output by nearest-neighbour coupling, similar to the tight-binding model of propagation in solid-state physics^31^. The coupling of *N* resonators creates *N* transmission resonances within each passband (see Supplementary Fig. 2). It is important to realize that each of the *N* resonators contribute via coherent superposition to each (Bloch) resonance, that is, these *N* resonances are the ‘supermodes’ of the combined structure, not *N* individual, uncoupled resonances. Figure 1a represents an idealized map of Ψ in terms of the quantity defined in the integrand of equation (1), that is, the product of the two-dimensional phase-matching points for *N*=5 coupled microresonators and the transfer functions at the two passbands of the generated photon pair. Along the horizontal and vertical axes, we have plotted the transmission spectra at the 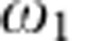 and 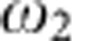 passbands, showing the five supermode transmission resonances. When the pump frequency is fixed, we obtain the equation 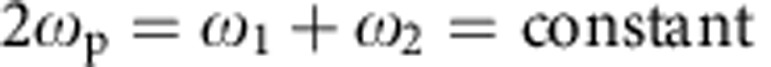, where 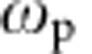 is the pump frequency and 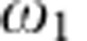 and 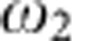 are the frequencies of the spontaneously generated photon pair. In the two-dimensional (2D) 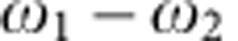 plane shown in Fig. 1a; this equation defines the JSI to be one of the diagonally oriented boxes shown with dotted white lines. Different choices of 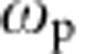 result in the selection of different regions and correspondingly different JSIs, for example, the regions marked by the labels ‘b', ‘c' and ‘d' correspond to the JSIs shown in Fig. 1b–d, respectively. (Other choices are also possible, corresponding to the unmarked diagonals in Fig. 1a).

The width (along the short axis) of the regions indicated by white dotted lines in Fig. 1a is given by the spectral width of the pump pulses, which in our experiment was less than 300 MHz and, therefore, only one narrow strip (one band of resonances) was selected. The length (along the long axis) of the regions indicated by white dotted lines in Fig. 1a is given by the lesser of two quantities: either the spectral extent of phase-matching (which was quite wide in our device, exceeding several nanometres^32^), or the extent of the transmission band, that is, the end-to-end span of the supermode resonances (which is determined by the strength of the inter-resonator coupling coefficient^33^). In our device, the latter quantity was the smaller one and, therefore, depending on the pump frequency, the number of peaks in the JSI could be varied from approximately one (in Fig. 1b, when the pump was positioned at supermode resonance located at the edge of its own transmission band) to the total number of resonators in the chain (5 in Fig. 1c, when the pump was positioned at the centre of its transmission band).

**Figure 2:**
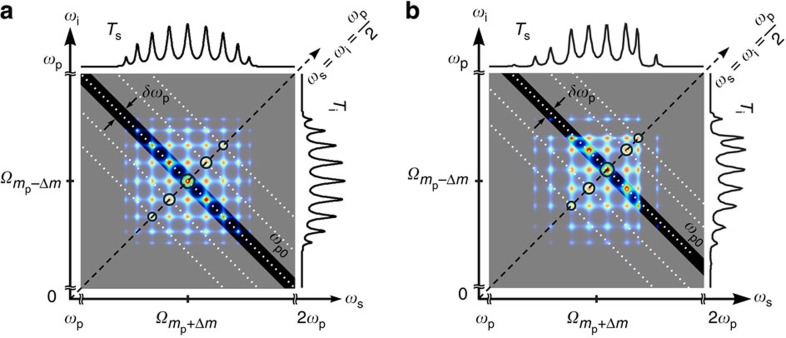
JSI. (**a**) The JSI can be interpreted as the section of the *N* × *N* array of phase-matching points in the 
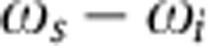
 plane which are selected by the input pump (of a particular energy 
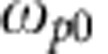
) based on the principle of energy conservation to lie along the diagonal regions shown by dotted white lines. The width of the selected region that defines the JSI is given by the spectral width of the pump envelope 
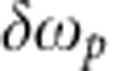
. The pump itself must be resonant with one of the supermodes in its transmission band; the possible choices of the pump frequency are shown by the black circles along the diagonal line, defined by 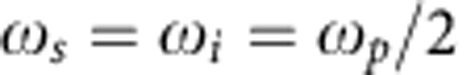. The JSI comprises from 1 upto *N* peaks; here *N* was taken as 11 in this representative calculation of the device used in the experiment, which consisted of 11 coupled silicon microring resonators. The horizontal axis represents the optical frequency of the ‘signal’ photon, and the vertical axis represents the optical frequency of the ‘idler’ photon; in both cases, the (ideal) transmission of one passband is shown to the top and right edges of the plot, respectively. (**b**) The experimentally measured transmission spectrum at the signal and idler passbands shows lower transmission at some of the band-edge resonances compared with the band centre, and a nonuniform spacing between the peaks because of fabrication disorder that affects the precise coupling coefficients between the resonators. Correspondingly, the calculated JSI shows about five peaks should have higher brightness than the others. Whereas all the peaks would be visible if the measurement process was noiseless, it is expected that the measured JSI in this device show between one and five peaks.

Figure 2a shows the construction of the JSI using the calculated transfer function of an 11-ring coupled-resonator structure, which models the device that was fabricated. The peaks are not of the same size and strengths because near the band edges, the resonances are sharper and the transmission magnitude decreases (see Supplementary Fig. 3). In recent work, Helt *et al.*^34^ have analysed the effect of loss in pair generation using SPDC where the fundamental and second-harmonic wavelengths are widely separated; a comparable analysis of loss in SFWM, where the pump, signal and idler wavelengths are much closer, remains to be developed. The JSI expected from the measured transmission at the signal and idler wavelengths is shown in Fig. 2b; this figure differs from the ideal because of at least two possible reasons: (i) the increased loss at band edges because of the increase in the slowing factor near the band-edge, (ii) errors in fabrication resulting in a different coupling coefficient than intended between the feeder waveguides and the first/last microring resonators (that is, imperfect apodization). Whereas these issues can be addressed with improved fabrication, here we expect that the JSI can be varied between one and five peaks. As discussed in Supplementary Note 2, the JSI can consist of any number of peaks ranging from 1 to *N* if the pump spectral width is narrow, and upto *N*^2^ if the pump spectral width can be changed. Chains of upto *N* =235 coupled silicon microring resonators have been demonstrated with an end-to-end spectral width of the transmission band of only ~5 nm^33^; however, those structures are not suitable for pair generation because, although the propagation loss per ring was quite low (~0.08 dB per ring), the total transmission loss was not low enough for such long chains.

### Schmidt number

We theoretically and experimentally test the factorizability of the generated two-photon states by performing a Schmidt decomposition. A bi-partite state is entangled if the number of non-zero eigenvalues is more than one. A widely used operational measure for entanglement is obtained by calculating the Schmidt number *K*, representing the number of orthogonal modes in the singular-value decomposition of the magnitude of the joint spectral amplitude (calculated here as the square root of the measured JSI under the assumption of flat spectral phase)^15^. The Schmidt number can equal the Shannon number for a communication system, which reflects the number of independent communication channels between source and receiver^35^ and is related to the ‘entanglement entropy’^16^. As shown in Fig. 1b–d, *K* can take on a wide range of values for the different JSIs. In the most general case, *K* can be continously varied by changing the inter-resonator coupling coefficients^36^; however, this is difficult to realize in practice. Here we show the selection of different values of *K* from a discrete set of alternatives, achieved by two different ways: either by changing the temperature of the chip, so that the transmission bands shift with respect to the (fixed) pump frequency, 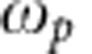, or alternatively, by changing the pump frequency while holding the chip temperature constant. In conventional SPDC experiments, the pump beam is shaped by bulk optics components in order to fine-tune the spectral correlations^12^,^17^,^25^,^26^; however, the ability to select manifestly different JSIs with vastly different *K*s is a unique aspect of this type of lithographically fabricated, multicomponent structure that has not been demonstrated before, for any other photon-pair source.

### Deconvolution of the filter point-spread function

**Figure 3:**
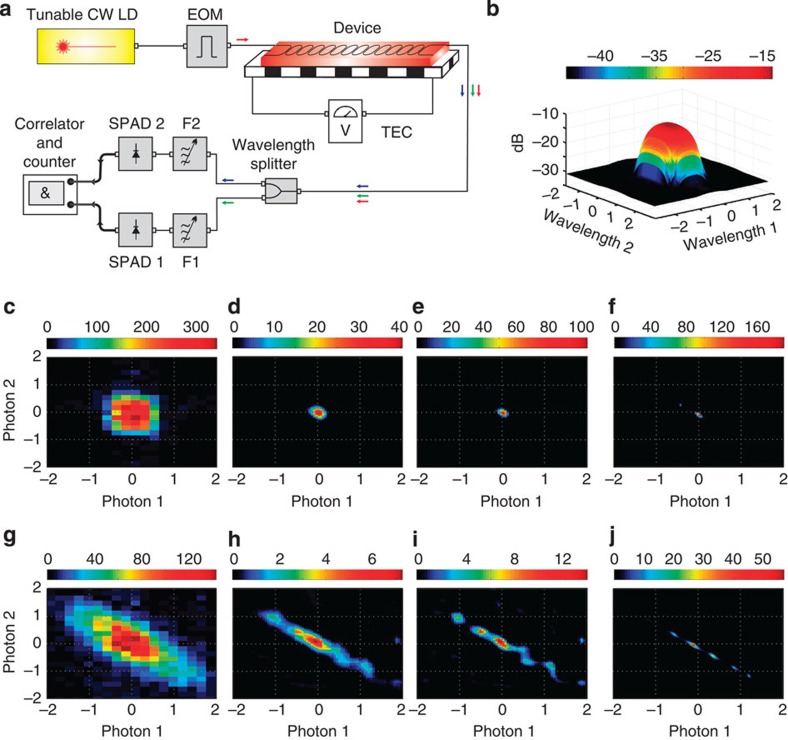
Deconvolution of JSI. (**a**) The optical pump was a tunable-wavelength continuous-wave (CW) laser diode (LD) modulated into pulses (~4 ns) using an electro-optic modulator (EOM). The device temperature was controlled using a thermoelectric controller (TEC). Tunable filters were used in front of the SPADs to measure the JSI. (**b**) The filter point-spread function (PSF) had a full-width at half-maximum of 0.6 nm along the axis for photon 1 and 1.0 nm along the axis for photon 2, which resulted in the measurement of a blurred JSI. The colourbar represents transmission in dB. (**c**,**g**) Examples of two different raw (blurred) JSIs, which were deconvoluted from the PSF using the iterative Richardson–Lucy algorithm, with (**d**,**h**) 20 iterations and with **e**,**i** 50 iterations. (**f**,**j**) A classical four-wave mixing experiment was performed to identify the phase-matching points, which form one component of the JSI expression equation (1). Each JSI was normalized to unit area, consistent with its definition as a probability density. In panels **c**–**j** the horizontal axes are in units of normalized wavelength (one unit equals 0.6 nm), measured relative to the respective band centres (‘Photon 1’: 1,548.8 nm, ‘Photon 2’: 1,578.7 nm).

Experimentally, there is no rapid measurement instrument for JSI at this time. The peaks and valleys that distinguish one JSI from another are separated by a frequency interval of ~20 GHz (only one-tenth of a nanometre), and whereas classical optical spectrum analysers are capable of providing such a high resolution, they are not sensitive at the single-photon level. On the other hand, quantum photon detectors such as single-photon avalanche diodes (SPADs) are not wavelength-selective. While 2D arrays of SPADs now being developed^37^,^38^ will be beneficial in the future, here, we use high-contrast tunable telecommunication-grade optical filters in front of the SPADs, as shown Fig. 3a to measure the JSI by scanning over the 2D frequency grid. The measured data, of which two examples are shown in Fig. 3c,g, represent the convolution of the actual JSI and the point-spread function of the filters shown in Fig. 3b. There are a number of different ways of deconvolving blurred images and as a representative method, we used the Richardson–Lucy (RL) algorithm^39^. The RL deconvolution is an iterative procedure for recovering, in a maximum-likelihood sense, a latent image that has been blurred by a known point-spread function. The end point of the iteration generally needs to be determined by the user, and we use our prior knowledge of typical JSIs (as shown in Fig. 2b) as a guideline, and confirm our choice by a classical four-wave mixing experiment^24^. In Fig. 3d,e, we show the results of deconvolution on the measured data (Fig. 3c) for 20 and 50 iteration steps, respectively. Similarly, for the measured data shown in Fig. 3g, we show the results of deconvolution with 20 and 50 iteration steps in Fig. 3h,i, respectively. In each case, we stopped at 50 iterations because the general shape and ‘sharpness’ of the JSI was then similar to the phase-matching function measured by a classical four-wave mixing experiment, shown in Fig. 3f,j for the two cases. The following discussion and insights do not depend on the exact stopping point of the de-blurring algorithm.

### Measurement of the JSI

**Figure 4:**
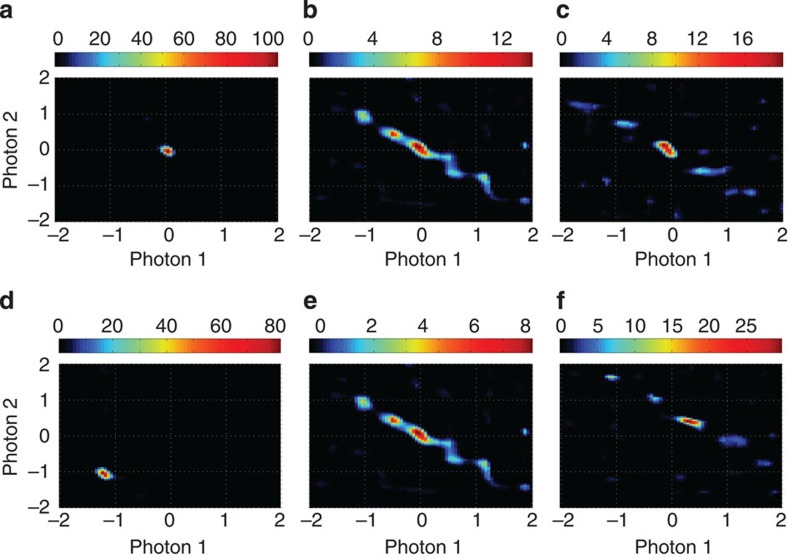
Experimentally generating photon pairs with different JSIs. Distinctly different JSIs can be obtained by tuning either the chip temperature or the pump wavelength. (**a**–**c**) Three JSIs measured with a fixed pump wavelength (1,563.61 nm) while tuning the TEC controlling the chip temperature to 27.7 °C (**a**), to 30.2 °C (**b**) and to 37.3 °C (**c**). (**d**–**f**) Three JSIs measured with a fixed TEC temperature setting of 30.2 °C while tuning the pump wavelength to be 1,563.03 nm (**d**), 1,563.61 nm (**e**) and 1,563.79 nm (**f**). In each case, the range of wavelengths over which data were acquired was the same. The Richardson–Lucy algorithm was used to deconvolve the point-spread function of the filters in front of the SPADs with 50 iterations. The Schmidt numbers are *K*=1.95 (**a**), 5.72 (**b**), 7.02 (**c**), 1.88 (**d**), 5.72 (**e**) and 5.47 (**f**). In each panel, the horizontal axes are in units of normalized wavelength (one unit equals a wavelength separation of 0.6 nm) measured relative to the respective band centre, which for ‘Photon 1’ was 1,548.8 nm and for ‘Photon 2’ was 1,578.7 nm. The vertical axes and colour scales are normalized so that the area under each JSI is unity, reflecting the fact that JSI is a probability density.

Figure 4 shows that different JSIs were obtained experimentally from the same photon-pair source. In Fig. 4a–c, the optical pump wavelength was kept constant at *λ*_p_=1,563.61 nm, and the chip temperature was tuned from 27.7 °C (Fig. 4a) to 30.2 °C (Fig. 4b) and to 37.3 °C (Fig. 4c). This range of temperature variations can be achieved by conventional thermoelectric controllers, such as those incorporated within commerical semiconductor lasers^1^. In Fig. 4d–f, the chip temperature was kept constant at 30.2 °C and the pump wavelength was tuned from 1,563.03 nm (Fig. 4d) to 1,563.61 nm (Fig. 4e) and to 1,563.79 nm (Fig. 4f). This range of wavelength variation required of the pump is comparable to the range of tunability offered in compact commercial tunable semiconductor lasers^1^, which can therefore be conveniently used to pump the silicon chip. Other different JSIs can also be obtained; however, we limit our report to these three examples because each frame shown in Fig. 4 took many hours to acquire since the optical filters were individually scanned over the 2D grid. Supplementary Note 3 and Supplementary Fig. 4 discuss a measurement example in which the de-blurring algorithm was not needed, for example, to decide between three distinct JSI alternatives, where the measurement took only 30 s. In Fig. 4a,c, the JSI showed a single peak, but because of the elliptical shape of the peak, the Schmidt number *K* is greater than 1. To reduce *K* without shaping the pump pulse, the device design should be adjusted to support a slightly broader spectral envelope for the pump pulse. The results of Fig. 4 show that temperature tuning and pump wavelength tuning result in similar effects, and either method of selecting different JSIs can be adopted in practice.

## Discussion

These results show the rich diversity of JSIs that can be generated by exploiting the degrees of freedom offered by chip-based lithographically fabricated photon-pair sources, as contrasted with their bulk crystal or fibre counterparts. In addition to the simple tuning schemes shown here, the individual resonator frequencies or coupling coefficients can be tuned to realize even more interesting JSI shapes.

Exploitation of higher-dimensional states for communication is of considerable research interest but the most widely studied examples of spatially encoded or polarization-encoded states are generally not robust to transmission over conventional optical fibre or realistic atmospheric channels. One example of utilizing the frequency degree of freedom to overcome these limitations is time-frequency coding^40^, where the ability to achieve high values of *K* without requiring a large frequency bandwidth can relax the timing constraints on detectors. Since there is a strong trade-off between detector speed and efficiency of single-photon detectors at the present time, the ability to generate strong spectral correlations over a narrow band has been desired^40^, so that the arrival-time coincidences can be stretched over a long enough period to be resolved by detectors. Unlike the elliptical JSIs generated by SPDC, the JSIs discussed here use the 2D frequency space more fully, and can achieve large Schmidt numbers in a bandwidth of only a few nanometres. More generally, using dimensionality as a general concept for quantum information was proposed by Hendrych *et al.*^41^ The experimental demonstration there relied on higher-dimensional multiplexing using orbital angular momentum, and strong frequency-entanglement may offer an alternative or additional degree of freedom.

In conclusion, these results have demonstrated control over the bi-photon spectrum using compact, chip-scale photon-pair sources, made using conventional planar lithography on silicon wafers with CMOS-compatible fabrication procedures. Such devices combine the high nonlinearity of the silicon material with internal degrees of freedom resulting from the design of the optical circuit in which the photons are generated. We have demonstrated that the JSI of the two generated photons can be controlled by either selecting different pump wavelengths, or different chip temperatures, and additional ways of programming the chip can be envisioned. The ability to control the JSI in real-time or through measurement-based feedback may lead to improved and more versatile semiconductor-based sources of quantum light for practical applications. The potential for high density of information encoding in the spectrum of the photon pairs will lead to advances in both regular and quantum optical communications.

## Methods

### Device design and fabrication

The devices were fabricated on silicon-on-insulator wafers at the Institute of Microelectronics in Singapore. Two etch steps were used for the rib waveguide cross-section. The waveguides, of width 0.55 μm, height 0.22 μm and slab thickness 70 nm, were designed for low-loss (~1 dB cm^−1^) transmission in the lowest-order mode of the transverse electric (TE) polarization defined relative to the device plane. Finite-element calculations showed three modes (two TE- and one transverse magnetic (TM)-polarized) that can be guided in straight waveguides; however, the TM mode and the high-order TE mode suffer higher bending loss in the microrings and do not propagate in the coupled-microring device. The microring resonators were arranged in a racetrack configuration with a radius of *R*=10 μm and directional coupler coupler length *L*_c_=10 μm. The rings were nominally identical, with interwaveguide gap in the directional couplers 0.3 μm. The propagation loss is defined as *α*=*α*_wg_*πR*_eff_/|*κ*|, in terms of the propagation loss of a silicon nanophotonic waveguide (*α*_wg_≈1 dB cm^−1^), the effective bending radius (*R*_eff_=*R*+*L*_c_/*π*) and the magnitude of the inter-ring coupling coefficient (|*κ*|≈0.36). Photon pairs were generated in a coupled-resonator waveguide consisting of 11 cascaded microrings cumulatively spanning a distance of 0.23 mm on the silicon chip between the input and output facets, excluding feeder waveguides.

### Experimental set-up

The experimental conditions for generating photon pairs and measuring coincidences were similar to our recent report^11^, showing good coincidence-to-accidental ratio for a wide range of temperatures and pump wavelengths. Light was coupled between optical fibres and the on-chip waveguides using inverted tapers on the waveguides, and lensed tapered single-mode polarization maintaining fibres. The insertion loss of each fibre-to-waveguide coupler was estimated as 3 dB, based on the calibration measurements on seperate test sites. Waveguides based on coupled resonators propagate light with a velocity smaller than that in conventional straight waveguides; the wavelength-dependent group index was measured to be between 24 and 40 (greater values at the shorter wavelengths^33^). The propagation loss was ~0.13 dB per ring including the slow-light enhancement of the loss. SFWM generated polarization-degenerate and frequency-nondegenerate photon pairs, and the wavelengths of the pump and the generated photons were in the telecommunication band near 1.55 μm. The required pump power was only ~0.2 mW after coupling to the on-chip waveguide from the input fibre; this value is considerably lower than that used in crystal-based SPDC sources and optical fibre-based SFWM sources of photon pairs. The pump wavelength was aligned with one of the transmission passbands of the coupled-resonator device near 1.55 μm. The continuous-wave pump light was carved into pulses of duration ~4 ns at repetition rate 60 MHz using an electro-optic modulator. For measurement, the photons were filtered using a tunable set of narrow band filters with full-width at half-maximum of 0.6 nm for the C-band and 1.0 nm for the L-band, insertion loss of 6 dB and passband-to-stopband contrast exceeding 150 dB. Single-photon counters (Micro Photon Devices) consisting of fibre-coupled InGaAs SPADs were used at a reverse bias voltage of 3.0 V and an estimated quantum efficiency of 10%. The SPADs were electrically gated from the same pulse generator as used to drive the optical modulator, with a trigger delay appropriately chosen based on the propagation time of light through the chip and connecting fibres. The average detector dark count rates in the two channels were measured to be 1.53 and 2.5 kHz. The electrical ‘clicks’ from the SPADs were processed using a fast AND gate (7,400 series TTL logic integrated circuit) and a frequency counter to measure the number of coincidences in a given time window. In Fig. 4, the peak coincidence rates are estimated as (from panels Fig. 4a–f in sequence): 72, 30, 18, 61, 30 and 20 kHz. These rates are calculated from the measured coincidence rates at the detectors after factoring out the chip-to-fibre coupling loss (3 dB), off-chip filter losses (6 dB) and detector quantum efficiencies (10%).

## Author contributions

R.K., J.R.O. and M.S carried out the experimental measurements and data processing. J.R.O. designed the device. M.S. performed the theory of JSI. S.M. co-ordinated the project. All authors read and contributed to the manuscript.

## Additional information

**How to cite this article:** Kumar, R. *et al.* Controlling the spectrum of photons generated on a silicon nanophotonic chip. *Nat. Commun.* 5:5489 doi: 10.1038/ncomms6489 (2014).

## Supplementary Material

Supplementary Information Supplementary Figures 1-4, Supplementary Notes 1-3 and Supplementary References
