# Supplementary material for: Controlling the spectrum of photons generated on a silicon nanophotonic chip
Source: Nat Commun. 2014 Nov 20;5:5489. doi: 10.1038/ncomms6489 (PMC4263184; doi:10.1038/ncomms6489)
Supplement: Supplementary Information — Supplementary Figures 1-4, Supplementary Notes 1-3 and Supplementary References [file ncomms6489-s1.pdf]

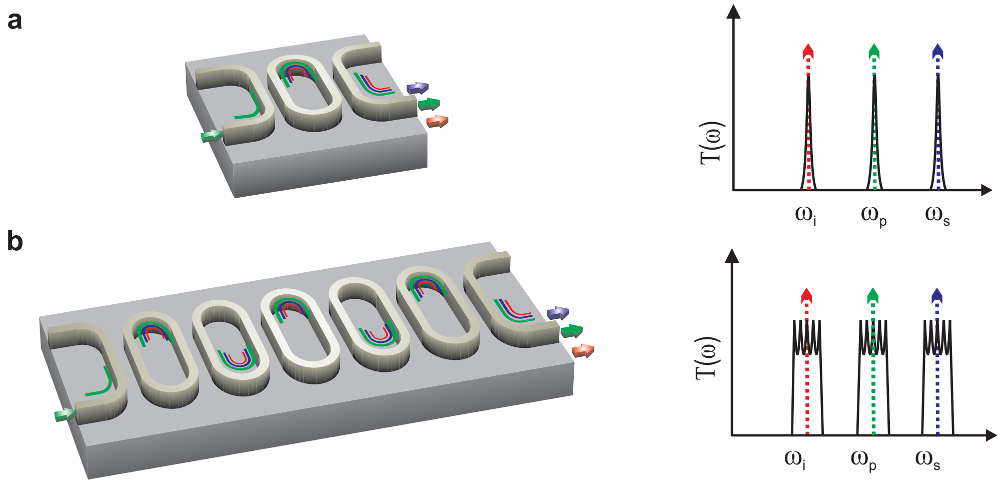

Supplementary Fig. 1. **Single and multi-resonator devices. a** A single microring resonator has one transmission peak in each passband, and the separation between the peaks is given by the free-spectral range (FSR) of the microring. The pump and the generated photons must be aligned to the (single) resonances as shown. **b** An N-resonator device (a representative example with $N = 5$ microrings is shown) has N transmission peaks in each passband. The pump and generated photons can be resonant with any of the N peaks, as along as energy conservation ($2\omega_p = \omega_i + \omega_s$) and phase matching ($2k(\omega_p) - k(\omega_s) - k(\omega_i) \approx 0$, where $k(\omega)$ is the modal propagation constant at the optical radian frequency ω) are satisfied.

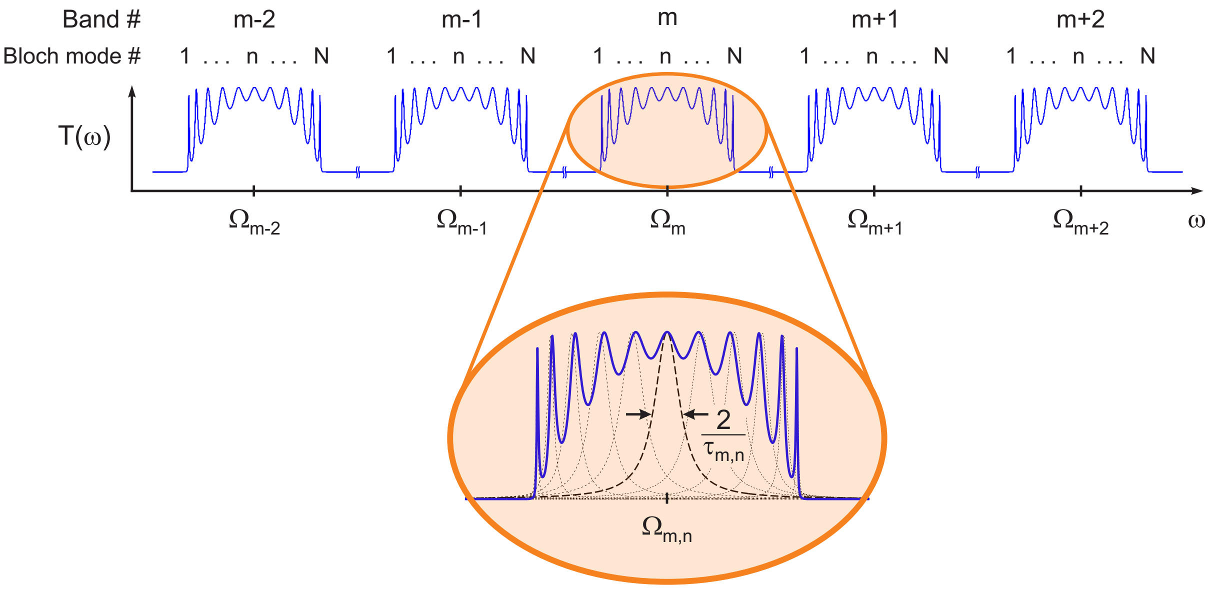

Supplementary Fig. 2. **Multi-peaked transmission spectrum.** The spectral transmission of a coupled-resonator device is written in Eq. (1) as the sum of individually-weighted Lorentzian lineshapes. The widths of the resonances are not uniform, and become narrower near the edges of the band, and result in the narrower widths of the peaks of the Joint Spectal Intensity at its edges, as shown in Fig. 2 (main paper).

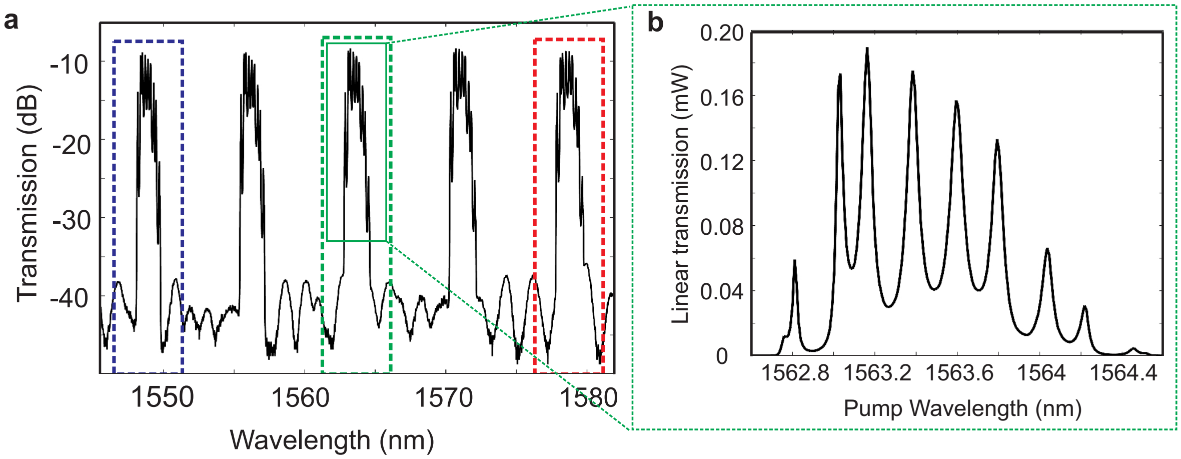

Supplementary Fig. 3. **Measured transmission spectrum.** **a**, Using a tunable diode laser and photodetector, the transmission of TE-polarized light through the coupled-resonator device was measured over several passbands. The bands indicated by the blue, green and red dashed-line boxes denote the passbands for the signal, pump and idler beams. **b** The transmission in a single passband is magnified, to show the individual transmission peaks (Bloch mode resonances). Unlike the idealized transmission calculated for an $N = 11$ coupled-resonator waveguide, shown in Fig. 2 and to the top and to the right of Fig. 2 (main paper), the transmission spectrum for the experimentally-fabricated device shows peaks of unequal height because of the enhanced loss near the band-edges and fabrication imperfections. Improved transmission characteristics can be obtained in coupled-microring devices with some alterations in the device design and in the fabrication procedure [1]. In this device, approximately five peaks near the center of the band stand out from the rest; therefore, the resulting JSI discussed in the main text is approximately that of an $N = 5$ coupled-resonator photon source.

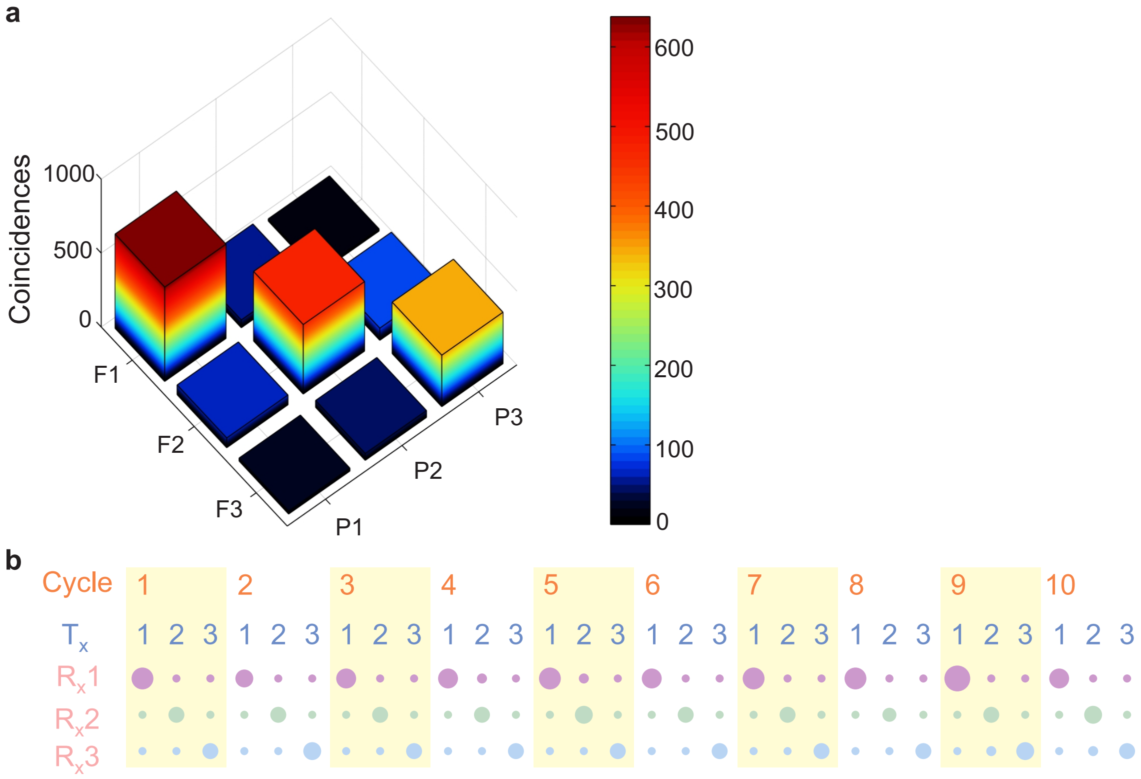

Supplementary Fig. 4. **Distinguishing between JSI's more rapidly a**, Photon pairs were generated in one of the three JSI's shown in panels Fig. 3d, 3e and 3f (main paper), and labeled P1, P2 and P3 respectively. By counting coincidences with three different filter settings (labeled F1, F2 and F3), the detector independently attempted to infer the JSI. Correct alignment, i.e., F1 to P1, or F2 to P2, or F3 to P3, resulted in the largest number of measured coincidences as shown by the tall bars on the diagonal, compared to the small number of mis-matched counts in the off-diagonal entries. The colorbar indicates measured coincidences in a duration of 150 s. **b**, The transmitter cycled through the "P1-P2-P3" pattern 10 times, each of duration 30 s. At the receiver, the diameter of the circles is proportional to the number of measured coincidences in the time interval. Since the diagonal entry in each case is the largest, the receiver can correctly identify which JSI was transmitted among the three alternatives within each time slot.

Supplementary Note 1: Coupled microring resonators

Microring resonators and closely-related device geometries such as the microdisk resonator, have been used recently for photon pair generation[2–6]. A generalization of the single-resonator device is shown in Supplementary Fig. (1). The transmission resonances in the passband of a coupled-resonator waveguide consisting of N resonators are split into N sub-peaks [7]. An optical input with narrow spectral width can propagate in any one of these modes, and similar to the single-resonator case, generates photon pairs by the principle of spontaneous four-wave mixing when the energy-conservation and phase-matching conditions are satisfied. The end-to-end width of the passband is determined by the inter-resonator coupling coefficient [8]. The width of each peak within the passband is taken as constant in Fig. 1 (main text) for simplicity; this assumption is relaxed in the discussion presented in the next section.

Supplementary Note 2: Joint Spectral Intensity

In a device consisting of N coupled resonators, the spectral intensity transmission $\mathcal{T}(\omega)$ shows N peaks in each passband, and these peaks play an important role in determining the JSI. For the purposes of this discussion, we write the transmission as a superposition of individually-weighted Lorentizian lineshapes, as shown in Supplementary Fig. 2,

$$\mathcal{T}(\omega) = \sum_{m=1}^{+\infty} \sum_{n=1}^{N} \mathcal{A}_{m,n} \mathcal{L}(\omega|\Omega_{m,n}; \tau_{m,n}) \tag{1}$$

where n indexes the resonators along the linear chain, and m is the azimuthal mode number, i.e., the number of wavelengths that physically fit along the resonator circumference. The value of m changes by 1 in going from one free spectral range (FSR) to the next.

The physical interpretation underlying Eq. (1) is that in each of the N Bloch modes that comprise the passband, the transmission is given by a Lorentzian line shape centered at $\Omega_{m,n}$ and with half-width at half-maximum (HWHM), $1/\tau_{m,n}$, defined as [9]

$$\Omega_{m,n} \approx \Omega_m - \frac{\Omega_m |\kappa|}{m\pi} \cos\left(\frac{n\pi}{N+1}\right) \tag{2}$$

$$\frac{1}{\tau_{m,n}} \approx \frac{1}{\tau_i} + \frac{2}{\tau_e} \frac{\sin^2\left(\frac{n\pi}{N+1}\right)}{\sum_{p=1}^{N} \sin^2\left(p\frac{n\pi}{N+1}\right)} \tag{3}$$

where $\Omega_m = (mc)/(n_{\text{eff}} R_{\text{eff}})$ is the passband center frequency for the optical mode of effective index n_{eff} circulating in a resonator of effective radius R_{eff}, $|\kappa|$ is the inter-resonator coupling coefficient, $1/\tau_{\text{i}}$ and $1/\tau_{\text{e}}$ are the internal and external dissipation rates, accounting respectively for the optical losses in the resonators, and for the additional losses arising from the input and output coupling.

In Eq. (1), each Lorentzian is weighted by a factor $\mathcal{A}_{m,n} = \exp(-S_{m,n}\alpha N\pi R_{\text{eff}})$, where α is the waveguide loss coefficient (units: dB/cm) of a conventional waveguide, and $S_{m,n}$ is the slowing factor, reflecting the fact that light at transmission band edges travels slower, and therefore experiences greater attenuation, than light at the band center. (S is defined as the group velocity in the constituent waveguide divided by the group velocity in the coupled-resonator waveguide[10, 11].)

Eq. (1) describes the transmission of optical beams as they propagate through the device. In the phenomenon of spontaneous four-wave mixing (SFWM), the interaction of an intense

optical beam with the vacuum fluctuations inside a medium with third-order nonlinearity leads to a process where two pump photons at frequency ω_p are simultaneously annihilated to create a pair of *Stokes* and *anti-Stokes* fields at ω_s and ω_i respectively. Assuming, for simplicity, no interactions other than SFWM[11, 12], (e.g., no cross-phase modulation or self-phase modulation), the interaction Hamiltonian is given by

$$H_{\rm int} \propto \iiint_{\mathcal{V}} d^3V S_s S_i \left(\frac{S_p+1}{2}\right)^2 \chi^{(3)} \hat{E}_p^{(+)} \hat{E}_p^{(+)} \hat{E}_s^{(-)} \hat{E}_i^{(-)} \tag{4}$$

where $\mathcal{V}$ is the interaction volume of the electric fields which are all assumed to be colinearly polarized (the waveguides are single mode and are designed to support the TE polarization only, defined with respect to the device plane), and nonlinearly coupled through the tensor $\chi^{(3)} = \chi^{(3)}_{xxxx}.S_j$ with $j = \{p, s, i\}$ are the slowing factors for the *pump*, *signal*, and *idler* respectively, according to the intra-cavity field enhancement factors.

Here we treat the intense pump field as a classical quantity and quantize the signal and idler fields, which is called the semi-classical assumption in quantum optics. The pump frequency ω_{p0} is chosen such that it corresponds to the eigenfrequency of a specific Bloch mode indexed by (m_p, n_p). The (complex) field of a strictly monochromatic pump at ω_{p0} propagating along the z-axis is

$$\hat{E}_p^{(+)} = \mathcal{E}_{p0}(x,y)\, e^{i(\omega_{p0}t - k_{p0}z)} \tag{5}$$

where $\mathcal{E}_{p0}(x,y)$ is the field modal distribution in the transverse plane, and k_{p0} is the propagation wave vector. It is understood that the real part of this expression should be taken in order to obtain the electrical field in the waveguide. In the experiment, the pump wave is modulated using an electro-optic modulator into approximately 4 ns pulses. To describe such a quasi-monochromatic pump, we introduce the spectral envelope $\Phi(\omega_p)$ which we shall assume, for simplicity, to be a Gaussian function of width $\delta\omega_p$, centered at ω_{p0},

$$\hat{E}_p^{(+)} \hat{E}_p^{(+)} = \mathcal{E}_{p0}^2(x,y) \int_{-\infty}^{+\infty} d\omega_p \Phi(\omega_p)\, e^{2i(\omega_p t - k_p z)}. \tag{6}$$

In view of Eq. (1), we write the signal and idler fields as

$$\hat{E}_j^{(-)} = \int_{-\infty}^{+\infty} d\omega_j \mathcal{E}_j(x,y) \sqrt{\mathcal{T}(\omega_j)} \mathrm{e}^{-i(\omega_j t - k_{m_j,n_j} z)} \hat{a}^\dagger(\omega_j), \tag{7}$$

where $j = \{s, i\}$, and $\hat{a}^\dagger(\omega_j)$ is the photon creation operator at ω_j. This representation explicitly accounts for the transmission through the coupled-resonator device of the generated photons, and assumes that the same transmission factor can be attributed to the

photons regardless of where physically along the chain the photons were created. (More formally, the nonlinear interaction populates a quantum state whose wavefunction, by definition, spans over all the resonators in the chain, since each Bloch waveform is given by a linear superposition over all resonators.)

The output bi-photon state is then obtained using first-order perturbation theory,

$$|\Psi\rangle = |0_s, 0_i\rangle - \frac{i}{\hbar}\int_{-\infty}^{+\infty} dt\, H_{\text{int}}|0_s, 0_i\rangle \tag{8}$$

which leads to a non-zero contribution given by

$$\begin{aligned}|\Psi\rangle \propto \iint_{-\infty}^{+\infty} d\omega_s d\omega_i\, S_s S_i \int_{-\infty}^{+\infty} d\omega_p \left(\frac{S_P+1}{2}\right)^2 \Gamma\Phi(\omega_p) \\ \times \left(\sum_{m_s=1}^{+\infty}\sum_{n_s=1}^{N} \mathcal{A}_{m_s,n_s}\mathcal{L}(\omega_s|\Omega_{m_s,n_s};\tau_{m_s,n_s})\right)^{1/2} \left(\sum_{m_i=1}^{+\infty}\sum_{n_i=1}^{N} \mathcal{A}_{m_i,n_i}\mathcal{L}(\omega_i|\Omega_{m_i,n_i};\tau_{m_i,n_i})\right)^{1/2} \\ \times \int_0^L dz\; e^{i\Delta kz}\; \delta(2\omega_p - \omega_s - \omega_i)\; |1_{\omega_s}, 1_{\omega_i}\rangle\end{aligned} \tag{9}$$

where $\Gamma = \iint dx\, dy\, \chi^{(3)}\mathcal{E}_{p0}^2(x,y)\mathcal{E}_s(x,y)\mathcal{E}_i(x,y)$ is the nonlinear overlap integral of the interacting fields with the material nonlinearity, and $\Delta k = k_{m_s,n_s} + k_{m_i,n_i} - 2k_p$ is the deviation from perfect phase matching. The Dirac delta function $\delta(2\omega_p - \omega_s - \omega_i)$ arises from the integration over time in Eq. (8) and demonstrates energy conservation: the interacting photons must strictly verify $2\omega_p = \omega_s + \omega_i$. Achieving the highest rates of pair generation requires that, despite the relatively short distance of propagation L in chip-scale devices compared to their bulk crystal or fiber counterparts, the phase matching condition, $\Delta k = 0$, should also be achieved.

From the dispersion relation of a coupled-resonator optical waveguide of finite length $L = N\Lambda = N\pi R_{\text{eff}}$, where Λ is the spatial periodicity along the axis of propagation, we can write down the discrete set of values which the wavenumbers can assume[9]. For the nth Bloch mode in transmission band m, we have

$$k_{m,n} = \frac{m\pi}{\Lambda} - \frac{\pi}{2\Lambda} + \frac{n\pi}{\Lambda(N+1)}. \tag{10}$$

Furthermore, we can write the frequencies ω_j for $j = \{p, s, i\}$ as

$$\omega_j = \Omega_{m_j,n_j} + \delta\omega_j = \frac{m_j c}{n_{\text{eff}} R_{\text{eff}}} - \frac{|\kappa| c}{\Lambda}\cos\left(\frac{n_j\pi}{N+1}\right) + \delta\omega_j \tag{11}$$

where $\delta\omega_j$ is the detuning with respect to the Bloch mode labeled (m_j,n_j), and the radial optical frequency of the pump is Ω_{m_p,n_p}. Thus, assuming that n_{eff} and $|\kappa|$ are constant within the frequency range of interest, the energy and momentum conservations are

$$\begin{aligned} m_p - m_s &= m_i - m_p = \Delta m \\ n_p - n_s &= n_i - n_p = \Delta n \\ \delta\omega_s &= -\delta\omega_i \end{aligned} \tag{12}$$

Hence, Eq. (9) becomes

$$\begin{aligned} |\Psi\rangle &\propto \iint d\omega_s d\omega_i\, S_s S_i \left(\frac{S_p+1}{2}\right)^2 \Gamma\Phi\left(\frac{\omega_s+\omega_i}{2}\right)\sqrt{\mathcal{T}(\omega_s)\mathcal{T}(\omega_i)}|1_{\omega_s},1_{\omega_i}\rangle \\ &\propto \iint d\omega_s d\omega_i\, S_s S_i \left(\frac{S_p+1}{2}\right)^2 \Gamma\Phi\left(\frac{\omega_s+\omega_i}{2}\right)\left[\sum_{\Delta m=0}^{m_p-1}\sum_{\Delta n=-\mathcal{N}}^{+\mathcal{N}} \mathcal{L}_-(\omega_s)\mathcal{L}_+(\omega_i)\right]^{1/2} |1_{\omega_s},1_{\omega_i}\rangle \end{aligned} \tag{13}$$

where $\mathcal{N} = \min(n_p-1, N-n_p)$ is the number of Bloch modes between the pump frequency and the closest band edge, and

$$\mathcal{L}_\pm(\omega_j) = \mathcal{A}_{m_p\pm\Delta m,n_p\pm\Delta n}\mathcal{L}\left(\omega_j|\Omega_{m_p\pm\Delta m,n_p\pm\Delta n};\tau_{m_p\pm\Delta m,n_p\pm\Delta n}\right) \tag{14}$$

From the integrand of Eq. (13), known as the joint spectral amplitude (JSA) [13, 14], we can express the joint spectral intensity (JSI), defined as JSI $= |\text{JSA}|^2$, which represents the probability of generating a pair of photons at frequencies (ω_s,ω_i),

$$\begin{aligned} \text{JSI}(\omega_s,\omega_i) &= \mathcal{K}\Phi^2\left(\frac{\omega_s+\omega_i}{2}\right)\mathcal{T}(\omega_s)\mathcal{T}(\omega_i) \\ &= \mathcal{K}\Phi^2\left(\frac{\omega_s+\omega_i}{2}\right)\sum_{\Delta m=0}^{m_p-1}\sum_{\Delta n=-\mathcal{N}}^{+\mathcal{N}} \mathcal{L}_-(\omega_s)\mathcal{L}_+(\omega_s) \end{aligned} \tag{15}$$

where $\mathcal{K}$ is a normalization constant such that $\iint d\omega_s d\omega_i\, \text{JSI}(\omega_s,\omega_i) = 1$. As can be seen from the summation terms [and shown in Fig. 2 (main paper)], the JSI contains $2\mathcal{N}+1$ peaks which satisfy energy and momentum conservation, ranging from a minimum of 1 to a maximum of N, the number of resonators in the chain [9, 15]. The peaks are not of the same size and strengths, since the underlying Lorentzian lineshapes shown in Supplementary Fig. 2 have different widths and are multiplied by different amplitudes, as described in Eq. (1).

If the pump spectral width can be increased, e.g., by shaping the pump pulse[16], the maximum number of JSI peaks that can be accessed increases from $2\mathcal{N}+1$ to N^2. In this

case, the pump pulses must be spectrally wide enough to excite all the resonances within a passband shown in Fig. 2, and correspondingly, the generated photon pairs also span their respective passbands. To achieve this experimentally will be challenging, and will requires a careful balance between the device group velocity dispersion, propagation length and spectral separation between the pump, signal and idler wavelengths.

Supplementary Note 3: Distinguishing between different JSI's more rapidly

At present, due to detector limitations as well as the un-optimized coupling between the chip and optical fibers which incurs additional losses, the acquisition of a complete JSI followed by computational de-blurring of the filter point-spread-function as shown in Fig. 3 (main paper) is a slow process. For some applications, it may not be necessary to wait for the full acquisition, e.g., a choice between a finite number of JSI alternatives can be made significantly faster.

In Supplementary Fig. 4, we show the results of a representative experiment in which the silicon chip transmits photon pairs with different JSI's encoded in different time slots, thereby encoding information in the quantum spectrum of the photon pair. We choose the three alternatives for JSI shown in Fig. 3d, 3e and 3f (main paper) as the possible choices at the transmitter. The receiver, which consists of the tunable filters and SPADs, measures a wide two-dimensional wavelength spectrum sufficient to cover all possible choices of the JSI. In the interest of making a faster measurement, we did not measure of the full 2.4 nm x 2.4 nm span shown in Fig. 3 (main paper) and limited our measurements instead to a smaller 0.6 nm x 0.6 nm window which was sufficient to distinguish between the three particular JSI's under consideration.

The tall coincidence peaks shown in Supplementary Fig. 4a correspond to the cases where the filter settings at the detector were correctly matched to the JSI that was transmitted. Coincidence counts were accumulated for a duration of 150 s for each of the 9 entries. The low off-diagonal peaks represent cases where the detector's JSI measurement was not matched to that of the transmitted JSI.

The dominant diagonal entries of the matrix of values showed that measurement over 150 s was sufficient to clearly distinguish at the receiver which JSI was transmitted. A further speed up can be achieved, to about 30 s. In Supplementary Fig. 4b, we show the results of a timed experiment, in which the pair-source was programmed to transmit the three JSI's shown in Fig. 3d, 3e and 3f (main paper) in sequence. In each case, the diagonal entry of the matrix was the dominant one, showing that the correct JSI could be identified.

Supplementary References

[1] Cooper, M. L., Gupta, G., Schneider, M. A., Green, W. M., Assefa, S., Xia, F., Vlasov, Y. A., and Mookherjea, S. Statistics of light transport in 235-ring silicon coupled-resonator optical waveguides. *Optics Express* **18**(25), 26505–26516 (2010).

[2] Clemmen, S., Huy, K. P., Bogaerts, W., Baets, R., Emplit, P., and Massar, S. Continuous wave photon pair generation in silicon-on-insulator waveguides and ring resonators. *Optics Express* **17**(19), 16558–16570 (2009).

[3] Azzini, S., Grassani, D., Strain, M. J., Sorel, M., Helt, L., Sipe, J., Liscidini, M., Galli, M., and Bajoni, D. Ultra-low power generation of twin photons in a compact silicon ring resonator. *Optics Express* **20**(21), 23100–23107 (2012).

[4] Davanco, M., Ong, J. R., Shehata, A. B., Tosi, A., Agha, I., Assefa, S., Xia, F., Green, W. M. J., Mookherjea, S., and Srinivasan, K. Telecommunications-band heralded single photons from a silicon nanophotonic chip. *Applied Physics Letters* **100**(26), 261104 (2012).

[5] Jiang, W. C., Lu, X., Zhang, J., Painter, O., and Lin, Q. Ultra-bright photon-pair generation on a silicon chip. In *Frontiers in Optics 2012/Laser Science XXVIII*, FW6C.10. Optical Society of America, (2012).

[6] Engin, E., Bonneau, D., Natarajan, C. M., Clark, A. S., Tanner, M., Hadfield, R., Dorenbos, S. N., Zwiller, V., Ohira, K., Suzuki, N., et al. Photon pair generation in a silicon micro-ring resonator with reverse bias enhancement. *Optics Express* **21**(23), 27826–27834 (2013).

[7] Mookherjea, S. and Yariv, A. Coupled resonator optical waveguides. *Selected Topics in Quantum Electronics, IEEE Journal of* **8**(3), 448–456 (2002).

[8] Cooper, M. L., Gupta, G., Schneider, M. A., Green, W. M., Assefa, S., Xia, F., Vlasov, Y. A., and Mookherjea, S. Statistics of light transport in 235-ring silicon coupled-resonator optical waveguides. *Optics Express* **18**(25), 26505–26516 (2010).

[9] Poon, J. K. and Yariv, A. Active coupled-resonator optical waveguides. I. gain enhancement and noise. *Journal of the Optical Society of America B* **24**(9), 2378–2388 (2007).

[10] Melloni, A., Morichetti, F., and Martinelli, M. Four-wave mixing and wavelength conversion in coupled-resonator optical waveguides. *Journal of the Optical Society of America B* **25**(12), C87–C97 (2008).

[11] Ong, J. R. and Mookherjea, S. Quantum light generation on a silicon chip using waveguides

and resonators. *Optics Express* **21**(4), 5171–5181 (2013).

[12] Chen, J., Li, X., and Kumar, P. Two-photon-state generation via four-wave mixing in optical fibers. *Physical Review A* **72**(3), 33801 (2005).

[13] Grice, W. and Walmsley, I. Spectral information and distinguishability in type-II down-conversion with a broadband pump. *Physical Review A* **56**(2), 1627 (1997).

[14] Yang, Z., Liscidini, M., and Sipe, J. Spontaneous parametric down-conversion in waveguides: a backward Heisenberg picture approach. *Physical Review A* **77**(3), 033808 (2008).

[15] Jeronimo-Moreno, Y., Rodriguez-Benavides, S., and U'Ren, A. B. Theory of cavity-enhanced spontaneous parametric downconversion. *Laser Physics* **20**(5), 1221–1233 (2010).

[16] Patera, G., Navarrete-Benlloch, C., de Valcarcel, G., and Fabre, C. Quantum coherent control of highly multipartite continuous-variable entangled states by tailoring parametric interactions. *The European Physical Journal D* **66**(9), 241 (2012).
